# Critical Role for P53 in Regulating the Cell Cycle of Ground State Embryonic Stem Cells

**DOI:** 10.1016/j.stemcr.2020.01.001

**Published:** 2020-01-30

**Authors:** Menno ter Huurne, Tianran Peng, Guoqiang Yi, Guido van Mierlo, Hendrik Marks, Hendrik G. Stunnenberg

**Affiliations:** 1 Department of Molecular Biology, Faculty of Science, Radboud University, 6525GA Nijmegen, the Netherlands

**Keywords:** embryonic stem cells, G1 checkpoint, P53, RB

## Abstract

Mouse embryonic stem cells (ESCs) grown in serum-supplemented conditions are characterized by an extremely short G1 phase due to the lack of G1-phase control. Concordantly, the G1-phase-specific P53-P21 pathway is compromised in serum ESCs. Here, we provide evidence that P53 is activated upon transition of serum ESCs to their pluripotent ground state using serum-free 2i conditions and that is required for the elongated G1 phase characteristic of ground state ESCs. RNA sequencing and chromatin immunoprecipitation sequencing analyses reveal that P53 directly regulates the expression of the retinoblastoma (RB) protein and that the hypo-phosphorylated, active RB protein plays a key role in G1-phase control. Our findings suggest that the P53-P21 pathway is active in ground state 2i ESCs and that its role in the G1-checkpoint is abolished in serum ESCs. Taken together, the data reveal a mechanism by which inactivation of P53 can lead to loss of RB and uncontrolled cell proliferation.

## Highlights

• The P53-P21 pathway is activated upon adaptation of ESCs to their pluripotent ground state.
• P53 is required for the elongated G1-phase characteristic to 2i ESCs.
• P53 binds the promoter and activates Rb1 expression.

## Teaser

Compared with somatic cells, mouse embryonic stem cells (ESCs) grown in serum-rich conditions display a shortened G1 phase and lack activity of the P53-P21 pathway. Stunnenberg and colleagues show that P53 is activated upon adaptation of ESCs to their pluripotent ground state in serum-free conditions. P53 modulates elongation of the G1 phase not via P21, but through transcriptional activation of the RB protein.

## Introduction

Mouse embryonic stem cells (ESCs) are pluripotent and self-renewing cells derived from the inner cell mass of the mouse blastocyst. ESCs can be indefinitely maintained *in vitro* in serum medium supplemented with the cytokine leukemia inhibitory factor (LIF) (Williams et al., 1988), hereafter called serum ESCs. In the past decade, new serum-independent culture conditions have been developed (Kolodziejczyk et al., 2015, Ying et al., 2008) giving rise to different flavors of ESCs that reflect different developmental states (Habibi et al., 2013, Marks et al., 2012). Mouse ESCs cultured in chemically defined 2i medium (N2B27 with PD0325901, CHIR99021, and LIF, hereafter called 2i ESCs) (Ying et al., 2008) were shown to have an unrestricted developmental potential and are therefore hypothesized to represent the ground state of pluripotency (Habibi et al., 2013, Marks et al., 2012).

The cell cycle of ESCs cultured in the presence of serum and LIF is extremely short, mainly due to truncated Gap- (G-) phases. The short G1 phase was considered to be characteristic of pluripotent mouse ESCs (Coronado et al., 2013). We have previously shown that the short G1 phase is characteristic of serum ESCs and is the result of ERK signaling. The latter pathway is inhibited in ground state pluripotent ESCs cultured in 2i, resulting in an elongated G1 phase (Ter Huurne et al., 2017). Proteins that delay G1 progression (e.g., the CDK2-inhibitors P21 and P27) are not expressed in serum ESCs (Marks et al., 2012, Savatier et al., 1994, Stead et al., 2002, Ter Huurne et al., 2017), but can be detected in 2i cultured G1-phase ESCs and contribute to the elongation of G1 phase. The combined knockout of P21 and P27 causes a decrease in G1-phase cells in 2i ESCs (Ter Huurne et al., 2017). P21 and P27 prevent CDK-mediated phosphorylation and inactivation of the pocket proteins, and thereby activate the G1 checkpoint.

Bypass of the G1 checkpoint in serum ESCs has been attributed to the lack of a P53-mediated DNA damage response (Aladjem et al., 1998, Dulić et al., 1994, Hong and Stambrook, 2004). The observation that P21, a prominent target of P53 in G1-arrest (Waldman et al., 1995), and a “readout” of P53 activity, is highly expressed in 2i and absent in serum ESCs suggests that the role or activity of P53 may be different (Ter Huurne et al., 2017). *In vivo* studies indicate that P53 is active in the inner cell mass (ICM) during early embryonic development (Goh et al., 2012) and by extrapolation in ground state pluripotent cells that are most reminiscent of ICM. These observations are in line with growing evidence that P53 plays an important role in embryonic development and differentiation. The exact role of P53 in ground state ESCs is, however, still unclear.

Therefore, we set out to decipher the distinct roles of P53 in ground state 2i and serum conditions. We generated a P53 knockout in an ESC cell line expressing the fluorescence ubiquitination cell cycle indicator (FUCCI) reporters that allow the designation of cells throughout the different phases of the cell cycle and subsequent analysis of specific populations. Our data show that P53 plays a critical role in G1-phase progression in ground state 2i, compared with serum ESCs. Moreover, genome-wide P53 binding and the transcriptome of P53^−/−^ 2i ESCs reveal that P53 directly regulates Rb1 expression in ground state ESCs, which affects the G1 phase.

## Results

### P53 Regulates G1-Phase Progression in 2i ESCs

**Figure 1:**
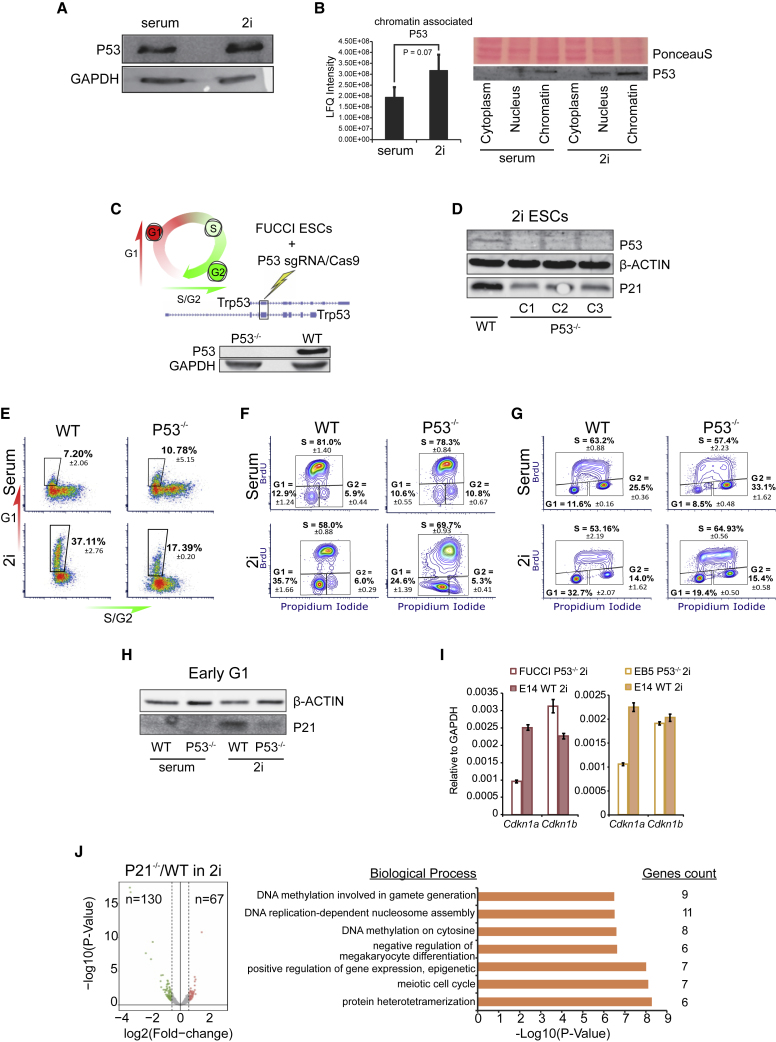
P53 Is Essential for the Elongated G1 Phase in 2i ESCs (A) Western blot (WB) of P53 level in total cell lysate of WT serum and 2i ESCs. (B and C) (B) Proteomics analysis and WB from different cellular fractions showing a higher level of chromatin-associated P53 protein in 2i ESCs compared with serum ESCs. Data from three biological replicates; significance was tested using an unpaired t test. (C) Cells in G1 phase expressing the FUCCI reporter are Kusabira Orange-positive, and cells in G2 phase are Azami Green-positive. Schematic representation of CRISPR/Cas9-mediated P53 knockout in FUCCI reporter ESCs targeting the common exon in *Trp53* isoforms, resulting in the absence of P53 as shown in the WB (P53^−/−^ clone 1). (D) Three independent P53^−/−^ clones were obtained, all showing a significant decrease in the expression of P21. (E) Analysis of FUCCI reporter expression in WT and P53^−/−^ cells. The longer G1 phase in 2i conditions (when compared with serum) is abbreviated in P53^−/−^ 2i ESCs, whereas in serum ESCs, no differences between WT and P53^−/−^ ESCs are observed. Reporter expression in P53^−/−^ clone 1 is shown (experiment performed in triplicate), which is representative for the three independent P53^−/−^ clones in at least two independent experiments. (F) Quantification of the different phases of the cell cycle in WT and P53^−/−^ (clone 1) FUCCI cells using BrdU incorporation combined with PI staining. Data from an experiment performed in triplicate that is representative for two independent clones. (G) BrdU/PI staining on WT and P53^−/−^ EB5 cells confirms the decrease in the percentage of G1-phase cells upon deletion of P53 in 2i conditions. Numbers indicate mean plus SD from a technical replicate. (H) WB showing decreased expression of P21 during G1 phase in P53^−/−^ FUCCI ESCs in 2i (P53^−/−^ clone 1). (I) RT-qPCR reveals a reduction in *Cdkn1a* mRNA levels in P53^−/−^ (clone 1) FUCCI ESCs and in the independent P53^−/−^ EB5 ESC line when compared with WT ESCs. No decrease in *Cdkn1b* mRNA was observed. Data from three technical replicates for each cell line are shown (bar graph shows mean and standard deviation). (J) Volcano plot showing transcriptome changes in P21^−/−^ G1-phase ESCs compared with the WT G1-phase ESCs cultured in 2i conditions. Each dot represents one gene. Significantly changed genes (adjusted p value <0.1 and a fold change of >1.5) are colored (downregulated genes in green and upregulated genes in red). GO clusters show the biological processes significantly enriched among the differential genes.

As a guardian of the genome, P53 minimizes the acquisition of DNA damage and plays a key role in maintaining genomic integrity in cells. A major pathway employed by P53 to prevent DNA damage is by halting G1-phase progression and S-phase entry via promoting *Cdkn1a* (coding for the P21 protein) expression, which results in the inhibition of the CYCLIN/CDK complexes (G. He et al., 2005). The elevated expression of P21 and elongated G1 phase in 2i ESCs (Ter Huurne et al., 2017) led us to hypothesize that P53 is active in 2i ESCs, but not in serum ESCs, and contributes to cell cycle regulation in the pluripotent ground state. Although the P53 protein level is surprisingly similar in these two ESC states (Figure 1A), the proteomic analysis of chromatin-associated (van Mierlo et al., 2019) and quantification of P53 protein levels in different cellular fractions indicated that the level of chromatin-bound P53 is slightly higher in 2i ESCs when compared with serum ESCs (Figure 1B).

To determine the effect of P53 on the cell cycle of ESCs, we created three independent P53^−/−^ clones in R1 ESCs that express the FUCCI reporter constructs using the CRISPR/Cas9 gene editing system. The single guide RNAs were designed to cut the longest common exon of different *Trp53* isoforms (Figure 1C). Upon deletion of P53, a clear reduction in P21 expression was observed in 2i cells (Figure 1D). Therefore, we asked whether the G1 phase in P53^−/−^ ESCs is perturbed due to the decrease of P21. FUCCI reporter analysis of the P53^−/−^ cells showed a dramatic decrease in the number of 2i ESCs in late G1 phase (Figure 1E). Serum ESCs enter S phase prematurely and therefore lack cells in late G1 phase. Accordingly, in P53^−/−^ serum ESCs, virtually no effect on the cell cycle was observed. Because the depletion of P53 has been reported to affect the level of geminin (Shen et al., 2012), we performed classical bromodeoxyuridine (BrdU)/propidium iodide (PI) staining in wild-type (WT) and P53^−/−^ FUCCI cells in parallel (Figure 1F). The results confirm that the percentage of G1-phase cells in 2i ESCs decreases upon deletion of P53. In addition, to verify that the observed phenotype is common to P53^−/−^ ESCs and not specific to FUCCI ESCs, we made use of an independent P53^−/−^ cell line (EB5, kindly provided by Hitoshi Niwa from Kumamoto University) to assess the distribution of cells over the different phases of the cell cycle using BrdU/PI staining. In 2i conditions, the number of cells in G1 phase was significantly lower in P53^−/−^ compared with WT ESCs, further confirming our previous observations (Figure 1G). Loss of P53 in serum ESCs had no measurable effect on the cell cycle (Figures 1E–1G). Because P21 is primarily expressed during G1 phase in 2i ESCs (Ter Huurne et al., 2017), the observed decreased expression in P53^−/−^ ESCs could be the result of the diminished number of cells in the G1 phase. A western blot on G1-phase sorted cells shows that the expression of P21 is lowered specifically in 2i G1-phase cells (Figure 1H). Our previous study showed that deletion of P21 is not sufficient to significantly shorten the G1 phase, but requires the deletion of both P21 and P27 (Ter Huurne et al., 2017); however, the *Cdkn1b* mRNA expression level was either slightly increased (R1-FUCCI P53^−/−^) or not affected (EB5 P53^−/−^ cells) (Figure 1I). Furthermore, transcriptome analysis on WT and P21^−/−^ G1-phase ESCs displayed only mild changes in the gene expression, with ∼130 were decreased and 67 were increased genes (Huang et al., 2008, Huang et al., 2009) (Figure 1J).

Taken together, we show that the P53 deficiency accelerates the G1 phase in 2i cells while no clear effect was observed in serum ESCs. Although P21 is downregulated upon deletion of P53, this alone is not sufficient to abbreviate the G1 phase, indicating that P53 may regulate the cell cycle in part independent of P21.

### Genes Involved in Cell Cycle Control Are Affected upon Deletion of P53 in 2i ESCs

**Figure 2:**
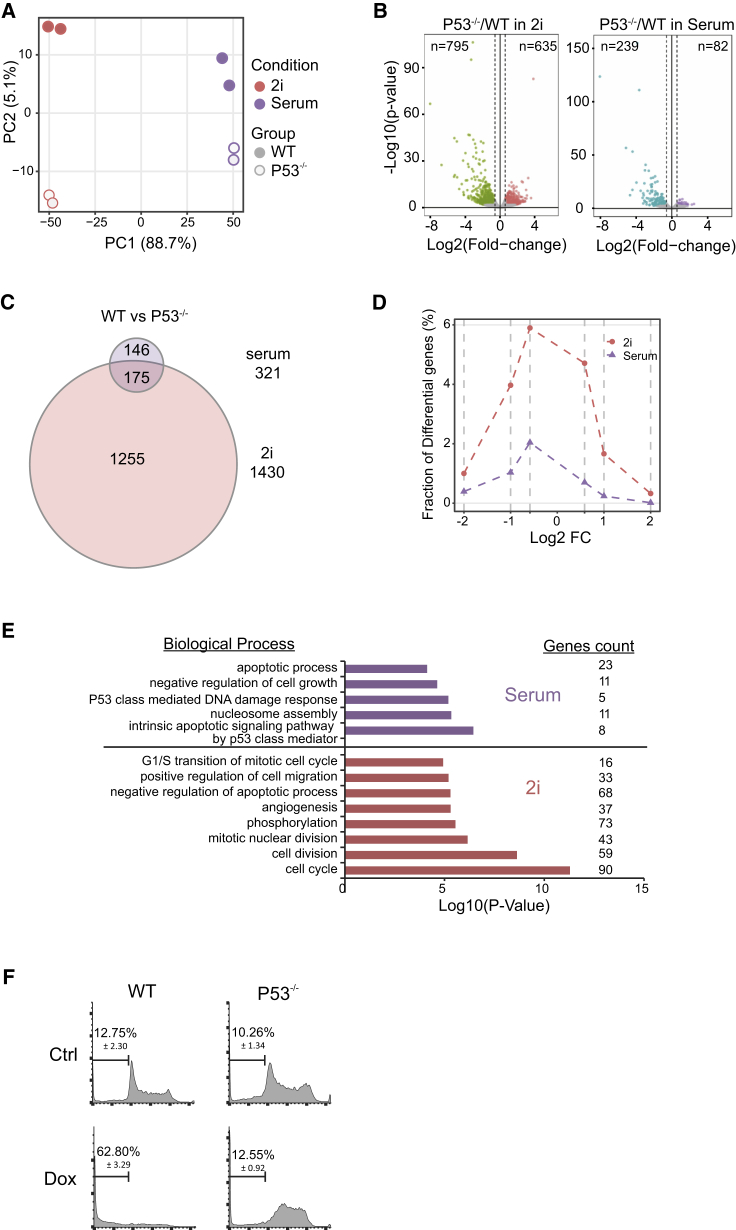
Genes Differentially Expressed in P53^−/−^ ESCs (A) Principal component analysis on gene expression of WT and P53^−/−^ G1-phase ESCs cultured in serum and 2i conditions. (B) Volcano plot showing the transcriptome changes in 2i or serum P53^−/−^ G1-phase ESCs compared with WT. Each dot represents one gene. Significantly changed genes (adjusted p value <0.1 and a fold change of 1.5) are colored (downregulated genes in green/blue and upregulated genes in red/purple). (C) Venn diagram showing the overlap of DE genes in serum and 2i. (D) Fraction distribution showing the fraction of genes differentially expressed between WT and P53^−/−^ ESCs cultured in either serum or 2i. (E) GO analysis revealing the significant biological processes in serum or 2i ESCs. Number of genes present for each category are indicated at the right. For the RNA-seq analysis, we made use of P53^−/−^ FUCCI (clones 1 and 2). (F) Serum WT and P53^−/−^ FUCCI (clone 1) ESCs treated with or without doxorubicin (1μM, 16 h), stained by PI and analyzed on fluorescence-activated cell sorting to assess apoptosis. Experiment performed in triplicate, numbers indicate mean percentage apoptotic ESCs (sub-G1 phase) plus SD. At least two independent experiments showed similar results.

To determine the impact of P53 depletion on the transcriptome of serum- and 2i ESCs, we carried out RNA sequencing (RNA-seq) on G1-phase sorted WT and P53^−/−^ ESCs in both culture conditions. The principal component analysis plot shows that the change in transcriptome between WT and P53^−/−^ is larger in 2i conditions compared with serum conditions (Figure 2A; PC2). Differential expression analysis identified 1,430 significantly differentially expressed (DE) genes in 2i WT versus P53^−/−^, while only 321 DE genes were found in serum WT versus P53^−/−^ (fold change >1.5, adjusted p value <0.1) (Figure 2B). Over half (175 of 321) of DE genes in serum were also found to be differentially expressed in 2i ESCs (Figure 2C). In addition, the fraction of genes differentially expressed between WT and P53^−/−^ was higher in 2i compared with serum (Figure 2D), suggesting a more extensive role of P53 in 2i conditions.

To gain a deeper understanding of the biological processes involving P53 in serum as well as in 2i, we performed gene ontology (GO) analysis on DE genes (Figure 2E). In both serum and 2i conditions, genes downregulated in the P53^−/−^ cells are enriched for apoptosis-related processes. In line with these findings, the loss of P53 prevents apoptosis in both serum and 2i conditions upon doxorubicin treatment (1μM, 16 h), as evident from the dramatic decrease in the number of cells in sub-G1 phase. In contrast to WT cells, the majority of P53^−/−^ cells stall in S and G2 phase after treatment with doxorubicin (Figure 2F for serum conditions; 2i conditions are not shown), which is in line with recent findings showing that the loss of P53 does not affect doxorubicin-induced G2/M arrest but can abolish apoptosis of both primed and naive-state ESCs (He et al., 2016). The analysis of the DE genes furthermore revealed that the set of genes differentially expressed in 2i conditions is most significantly enriched for genes involved cell cycle processes, which is not obtained for serum-cultured ESCs. Thus, genes involved in the cell proliferation are highly affected in the G1 cells of 2i ESCs due to loss of P53.

### P53 Activates *Rb1* to Elongate G1 Phase in 2i ESCs

**Figure 3:**
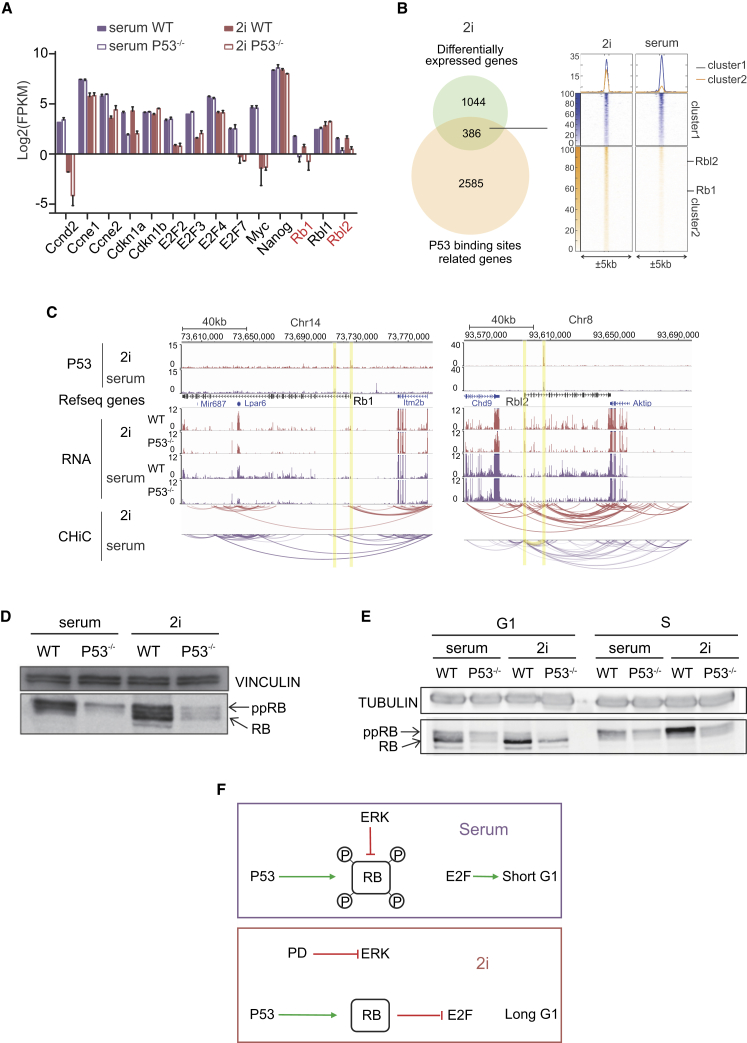
P53 Activates *Rb1* to Elongate G1 Phase in 2i ESCs (A) Bar graph showing differential expression of cell cycle and other regulators in serum and 2i cultured WT and P53^−/−^ ESCs during G1 phase. The expression of *Rb1*, *Rbl2*, and *Cdkn1a* (P21) is decreased in P53^−/−^ both in serum and 2i ESCs. (B and C) (B) A total of 2,585 genes are associated with P53 binding sites, 386 of these genes are expressed differentially in 2i P53^−/−^ compared with 2i WT cells. (C) ChIP-seq and Capture HiC indicate that P53 binds regions that locate or interact with the TSS of *Rb1* and *Rbl2*. (D) WB showing that the protein level of hyper-phosphorylated RB (ppRB) and hypo-phosphorylated RB (RB) is reduced in P53^−/−^ FUCCI ESCs (clone 1) in serum and 2i, respectively. (E) WB showing the protein level of RB throughout the cell cycle in P53^−/−^ (clone 1) and WT FUCCI ESCs in serum and 2i conditions. At least two independent experiments showed similar results. (F) Proposed model that displays the role of P53 and Rb1 in ESC cell cycle control.

The differences in cell cycle and transcriptome between P53^−/−^ and WT ESCs indicate that P53 is essential for the elongated G1 phase in 2i conditions compared with serum ESCs. Although there was a substantial decrease in the expression of P21 in the P53^−/−^ ESCs, the sole loss of P21 cannot explain the changes in the cell cycle (Ter Huurne et al., 2017). Besides *Cdkn1a* (P21), a range of genes involved in cell cycle regulation are differentially expressed between P53^−/−^ and WT in 2i-cultured cells (Figure 3A). To identify direct targets of P53 connected to the cell cycle control, we performed P53 chromatin immunoprecipitation sequencing (ChIP-seq) in serum and 2i ESCs. The number of P53 binding sites is significantly higher in 2i (3,595 versus 1,347 in serum), supporting our previous observations that P53 has a more prominent role in 2i conditions compared with serum conditions. Higher general chromatin accessibility due to differences in epigenetic make-up (e.g., the lowered level of DNA methylation in 2i conditions) (Habibi et al., 2013, Leitch et al., 2013), could contribute to the increased binding of P53 in 2i. To identify the genes that are likely under direct regulation of P53, we annotated the P53 ChIP-seq binding sites using HOMER (Heinz et al., 2010). The result shows that 386 genes with P53 binding in their promoters or enhancers are also differentially expressed in 2i P53^−/−^ ESCs (Figure 3B). Out of these 386 genes, 129 have a higher P53 ChIP-seq signal in serum (cluster 1), whereas 257 have a higher ChIP-seq signal in 2i (cluster 2).

Subsequent GO-term analysis revealed that this set of 386 genes was significantly enriched for genes that regulate cell cycle arrest (10 genes with a Benjamini corrected p value of 9.6 × 10^−3^). Interestingly, *Rb1* is among those genes. We found that in P53^−/−^ ESCs, the *Rb1* and *Rbl2* transcripts that encode RB and P130, respectively, are decreased compared with WT ESCs (Figure 3A). The pocket proteins RB and P130 are well known to be involved in the control G1-phase progression in 2i ESCs by inhibiting the activity of the E2F transcription factors (Ter Huurne et al., 2017) and are themselves downstream targets of the CDK/CYCLIN pathway. P53 ChIP-seq in 2i ESCs showed that P53 binds to the Transcriptional Start Site (TSS) and gene body of *Rb1* and to the gene body of *Rbl2*. Integrating analysis of RNA-seq, ChIP-seq, and Capture HiC data (Joshi et al., 2015) revealed that the P53 binding sites interact with the TSS of *Rb1* and *Rbl2*, suggestive of a direct transcriptional regulation (Figure 3C).

Although the expression of these pocket proteins was drastically reduced both in serum as well as in 2i conditions after P53 knockout, inactive hyper-phosphorylated RB was the predominant form in serum ESCs, whereas hyper-, as well as active hypo-phosphorylated RB, could be detected in 2i ESCs (WT was shown previously [Ter Huurne et al., 2017]) (Figure 3D). Since the cell cycle distributions of asynchronously growing WT and P53^−/−^ 2i ESCs are different, we next determined RB protein levels throughout the cell cycle in WT and P53^−/−^ serum and 2i ESCs. The result showed a clear reduction of RB expression in P53^−/−^ ESCs and furthermore showed that the fraction of hypo-phosphorylated RB was higher in 2i than in serum G1 cells (Figure 3E).

Taken together, our data strongly suggest that P53 directly activates the transcription of the pocket proteins RB and P130, thereby elongating G1 phase in 2i ESCs. Due to increased ERK-/CDK/CYCLIN-signaling, RB is constitutively hyper-phosphorylated in serum ESCs and the deletion of P53 has a minor effect in these cells (Figure 3F).

## Discussion

Rapid proliferation is a hallmark of pluripotent stem cells and has intrinsically been associated with their unique cell cycle (Vallier, 2015). A truncated G1 phase is fundamental to the cell cycle of ESCs and is reflected by their smaller size when compared with somatic cells (Hindley and Philpott, 2013). The shortened G1 phase is accompanied by the impairment of pathways that control genomic integrity during G1 phase in serum ESCs (Hong et al., 2007). However, the long-standing notion that ESCs have a shorter G1 phase compared with somatic cells was mainly based on studies performed in serum-cultured ESCs (Ballabeni et al., 2011, Stead et al., 2002). Recently, we and others have shown that 2i ESCs have a much longer G1 phase compared with serum ESCs (Cannon et al., 2015, Ter Huurne et al., 2017). How these differences in cell cycle between serum and 2i ESCs affect the biological processes that take place during G1 phase has remained unclear.

P53 is well known for its pivotal role in induction of G1-arrest to protect genomic integrity. Early studies in serum ESCs have found that although P53 is highly expressed, it cannot act as a regulator of G1 phase progression due to functional uncoupling of the P53/P21 axis (Suvorova et al., 2016). The elevated expression of P21 and the identification of the elongated G1 phase led us to hypothesize that the P53/P21 pathway may be activated and extend the G1 phase upon transition of serum ESCs to 2i conditions. The results presented here indicate that P21 expression is indeed regulated by P53. Unexpectedly, however, the elongated G1 phase in 2i ESCs depends on a novel unexplored function of P53 in the cell cycle of ESCs. In serum ESCs, the pocket proteins that inhibit G1-phase progression are lowly expressed and inactivated due to abundant ERK signaling (Ter Huurne et al., 2017). In 2i ESCs, the ERK signaling pathway is, however, inhibited, leading to the activation of the RB-mediated G1 checkpoint. Our results imply that P53 regulates not only the expression of P21, but also the expression of the downstream pocket proteins RB and P130. Since these proteins are not active in serum ESCs, this function of P53 has not been observed in serum ESCs (Figure 3F). By regulating the expression of the pocket proteins, P53 is crucial for the elongated G1 phase in 2i conditions. The loss of P53 does, however, not fully shorten the G1 phase in 2i ESCs to the level of that in serum ESCs, which suggests that other mechanisms are involved as well in regulation of G1-phase progression in 2i ESCs. The lowered ERK signaling and reinstatement of the P53-mediated G1 checkpoint in 2i ESCs suggests that these cells are better able to cope with DNA damaging events, which, however, remains to be shown.

P53 is highly expressed in the early embryo, but its functional role is still elusive. Our findings suggest that in the early embryo where ERK signaling is absent, P53 plays a critical role during G1 phase to restrict rapid cell proliferation by modulating the expression of the pocket proteins. Interestingly, the cellular senescent state that resembles diapause *in vivo* depends on the presence of this family of proteins (Scognamiglio et al., 2016, Ter Huurne et al., 2017). Our observations, therefore, suggest that P53 plays a role in diapause. Possibly, P53 is highly expressed in early rodent embryos in order to induce diapause in response to stressful conditions.

Besides the differences in cell cycle control between WT and P53^−/−^ 2i ESCs, the RNA-seq data uncovered a large number of developmental genes (among others involved in angiogenesis and the development of the nervous system) that are negatively affected by loss of P53 in 2i conditions. These findings are in line with previous reports that suggest an important role for P53 in differentiation, and imply that P53 is functionally more dynamic in 2i. How P53 regulates the expression of developmental genes in 2i remains to be determined, possibly the differential regulation of the pocket proteins plays a role, considering their role in development and differentiation (Calo et al., 2010, Julian and Blais, 2015).

Altogether, we show that in ESCs, the function of P53 differs depending on the cellular state. In ground state 2i ESCs, P53 is involved in controlling the cell cycle via directly regulating the expression of the pocket proteins.

## Experimental Procedures

### Cell Culture

Mouse ESCs were cultured in serum and 2i conditions, as described previously (Ter Huurne et al., 2017). Media were refreshed every day and cells passaged every 3 days.

### Immuno Blotting

Immunoblotting was performed as described previously (Ter Huurne et al., 2017). Details and information on antibodies can be found in the Supplemental Experimental Procedures.

### Genome Editing Using CRISPR-Cas9

The CRISPR-Cas9 gene editing technology was used to knock out Trp53 (P53), as described previously (Ter Huurne et al., 2017). In brief, FUCCI ESCs were transfected with the CRISPR-Cas9 plasmid containing a guide RNA using lipofectamine-3000. Cells expressing GFP over background were single cell sorted, and approximately 7 days thereafter, colonies were picked for expansion.

### Flow Cytometry

For cell cycle analysis, cells were prepared as described previously (Ter Huurne et al., 2017) and subsequently analyzed using a FACScalibur or FACSverse flow cytometer (Becton Dickinson). The BD FACS Aria cell sorter was used to sort FUCCI ESCs from different phases of the cell cycle. Cells without reporters and cells without BrdU incorporated were used as negative controls to set the gates.

### qRT-PCR and RNA-seq

Total RNA were extracted using the RNeasy Mini Kit following the manufacturer's protocol. Reverse transcriptase and random primers (p(dN)_6_) or Oligo(dT)_12-18_ primers were used for reverse transcription. Real-time qPCR was performed using the SYBR Green Supermix. Gapdh primers (Fwd: TTCACCACCATGGAGAAGGC, Rev: CCCTTTTGGCTCCACCCT) were used to normalize the expression. P21 and P27 primers have been described before (Teratake et al., 2016). RNA-seq sample prep and analysis can be found in the Supplemental Experimental Procedures.

### ChIP-seq

ChIP-seq samples were prepared as described previously (Ter Huurne et al., 2017). In brief, ESCs were fixed using 1% formaldehyde for 10 min at room temperature. After quenching using 1.25M glycine, cells were lysed using 1% SDS and sonicated then diluted in 1x PBS containing 0.5% Bovine Serum Albumin. Diluted chromatin containing 30μg DNA was incubated with 15μg P53 antibody and 60μL pre-blocked beads at 4°C overnight. After subsequent washing steps using TE buffers, beads were eluted in 200 μL elution buffer (1% SDS, 0.2M NaCl, 0.1μg/μL Proteinase K) at 65°C thermo shaker 1000 rpm for 20 min. Supernatants were purified with MinElute PCR Purification Kit; 1 to 5 ng of DNA was used for library construction with the KAPA Hyper Prep Kit. Details and information can be found in the Supplemental Experimental Procedures.

## Author Contributions

M.t.H., T.P., and H.S.G. conceived the study and wrote the manuscript. M.t.H. and T.P. performed experiments. M.t.H., T.P., and G.Y. analyzed the data. G.v.M. generated and analyzed the proteomics data. H.M. contributed to the study design and helped draft the manuscript. Funding was obtained by H.G.S.

## Accession Numbers

The accession number for the RNA-seq and ChIP-seq data in this paper is GEO: GSE141179.

## Supplemental Information

Document S1. Supplemental Experimental Procedures and Tables S1 and S2

Document S2. Article plus Supplemental Information
