# Supplementary material for: Critical Role for P53 in Regulating the Cell Cycle of Ground State Embryonic Stem Cells
Source: Stem Cell Reports. 2020 Jan 30;14(2):175–83. doi: 10.1016/j.stemcr.2020.01.001 (PMC7013234; doi:10.1016/j.stemcr.2020.01.001)
Supplement: Document S1. Supplemental Experimental Procedures and Tables S1 and S2 [file mmc1.pdf]

Stem Cell Reports, Volume *14*

Supplemental Information

Critical Role for P53 in Regulating the Cell Cycle of Ground State Embryonic Stem Cells

Menno ter Huurne, Tianran Peng, Guoqiang Yi, Guido van Mierlo, Hendrik Marks, and Hendrik G. Stunnenberg

Supplemental Experimental Procedures

Genome editing using CRISPR-Cas9

We made use of the CRISPR-Cas9 gene editing technology to knock out Trp53 (P53). An online tool (crispr.mit.edu and Benchling) was used to design the gRNAs that were cloned into the plasmid Cas9(BB)-2A-GFP (Addgene plasmid 48138) as described previously (ter Huurne et al., 2017). FUCCI serum ESCs were transfected using lipofectamine-3000 (Thermofisher # L3000015). After 48 hours, cells expressing GFP over background (from the Azami Green reporter) were sorted with a BD FACS Aria. Cells were split at clonal density and after approximately 7 days colonies were picked for expansion. Genomic DNA from individual clones was extracted using the Wizard Genomic DNA extraction kit. The targeted region was PCR amplified and Sanger Sequenced. gRNA oligonucleotides were as follows:

Trp53_gRNA_a_Fwd	CACCGGAGCTCCTGACACTCGG
Trp53_gRNA_a_Rev	AAACCCGAGTGTCAGGAGCTCC

Immuno blotting

Cells were trypsinized and washed with PBS. Cell pellet were lysed in RIPA buffer (150mM NaCl, 1% NP-40, 0.5% NaDOC, 0.1% SDS, 50mM Tris-HCl pH=8) with fresh EDTA-free protease inhibitor cocktail (Roche #4693132001). Protein concentration was measured using Bio-rad protein assay (#500-0006). Cell extracts were loaded equally and separated by 7-12% SDS-PAGE, electrotransferred to nitrocellulose membranes and incubated in blocking buffer (5% nonfat milk in TBST) for 1 hour at room temperature. Membranes were incubated with primary antibody (1:1000 diluted in blocking buffer) over night at 4°C then washed 5 times in TBST for 5 minutes at room temperature and incubate with second antibody (1:2000 diluted in blocking buffer) at room temperature for 1 hour. After five washes with at room temperature for 5 minutes ECL substrate (ThermoFisher #32106) was added and images were acquired. The primary antibodies used in this study are P53 (Oncogene #OP03), P21 (SantaCruz #sc-6546), RB (BD Bioscience #554136), GAPDH 6C5 (Abcam #8245), TUBULIN (SantaCruz #sc5286), VINCULIN (SantaCruz #sc5573) The secondary antibodies used are Swine anti-Rabbit HRP (Dako #P0217), Rabbit anti-Rat HRP (Dako #P0450) and Rabbit anti-Mouse HRP (Dako #P0161).

qRT-PCR and RNA-seq

Total RNA were extracted using the RNeasy Mini Kit (Qiagen #74106) following the manufacturer's protocol. SuperScript^{TM} III Reverse Transcriptase (ThermoFisher #18080093) and random primers ($p(dN)_6$, Roche #11034731001) or Oligo$(dT)_{12\text{-}18}$ primer were used for reverse transcription. Real-time qPCR was performed using the iQ™ SYBR® Green Supermix (Bio-Rad #1725006). An endogenous control (Gapdh; primers Forward: TTCACCACCATGGAGAAGGC, Reverse: CCCTTTTGGCTCCACCCT) was used to normalize the expression. Biological replicates were performed for all RT qPCR reactions. P21 and P27 primers have been described before (Teratake et al., 2016).

For RNA-Seq, 5 µg of extracted RNA was depleted from ribosomal RNA using Ribo-Zero Gold Kit (Epicentre Madison, #MRZG126). After fragmentation of the rRNA-depleted RNA, 500ng was reverse-transcribed using SuperScript™ III Reverse Transcriptase and random primers $(dN)_6$ following the manufacturer's instructions. Next, libraries were prepared using the KAPA Stranded RNA-Seq Library Preparation Kit (KAPA #8400) following the manufacturer's instructions.

ChIP-seq

ESCs were fixed using 1% formaldehyde (Millipore #344198) for 10 min at room temperature then quenched by adding 1.25M glycine to a final concentration of 0.125M. Cell pellets were snap frozen and stored in -80°C. Every 10 million cells were lysed by 300µL 1% SDS include freshly made Protease Inhibitor (PI) cocktail (Roche #4693132001), sonicated with Diagenode Bioruptor Pico and diluted with 2.7mL PBA (1x PBS + 0.5% BSA) with fresh added PI cocktail. Dynabeads protein A+G (Invitrogen #10008D, 10009D) were washed and pre-blocked with cold PBA for 30 minutes. Diluted chromatin containing 30µg DNA was incubated with 15µg P53 antibody (Novocastra #CM5P) and 60µL pre-blocked beads and rotated at 4°C overnight. After incubation, beads were washed subsequently with High-NaCl, Low-NaCl, LiCl, TE and TE washing buffers for 10 minutes and transferred to new eppendorf tube. Beads were eluted with 200µL elution buffer (1%SDS, 0.2M NaCl, 0.1µg/µL Proteinase K) at 65°C thermo shaker 1000rpm for 20 minutes. Supernatants were purified with MinElute PCR Purification Kit (QIAGEN #28006). 1-5ng of DNA was used for library construction with KAPA Hyper Prep Kit (KAPA #KK8504).

ChIP-seq analyses

For each sample, all 42bp reads were mapped onto the mouse genome (mm9) using Burrows-Wheeler Aligner (BWA) aligner (Li and Durbin, 2009) with default parameters. The mapped reads were regarded as input for Picard

Mark Duplicates (http://broadinstitute.github.io/picard/) to remove potential PCR duplicates. MACS2 (Zhang et al., 2008) was used to call P53 peaks with a narrow q-value cut-off of 0.01. Read density profiles are displayed as fold enrichment track generated by normalizing ChIP data over input DNA pileup signal files using MACS2. These profiles were further visualized by deepTools (Ramírez et al., 2016)..

RNA-seq analyses
RNA sequencing reads were aligned to the mouse reference genome mm9 using STAR tool (Dobin et al., 2012) which could enumerate gene-level read counts at the same time. The differentially expressed genes were identified with the DESeq2 package (Love et al., 2014) by comparing knockout with wild-type groups. Only those genes greater than 1.5-fold changed at Benjamini-Hochberg-corrected P-value < 0.1 were considered significantly deregulated. The transcriptional levels of genes were estimated as Fragments Per Kilobase per Million aligned reads value (FPKM) values using by Cufflinks (Trapnell et al., 2010).

Dimensionality reduction and functional annotation
To explore potential variances between different groups, we performed principal component analysis. Top 3,000 variable genes were first selected based on interquartile range (IQR) of normalized gene expression levels, and further used to reduce dimensionality of the dataset by pca function in R. We used the DAVID tool (Huang et al., 2008) to assess enriched gene ontology terms and pathways in order to gain insight into the biological functions for deregulated genes. Only terms with Benjamini-adjusted P-value < 0.05 were considered significantly overrepresented.

Table S1: Antibodies used in this study
<u>Immunoblotting</u>

Name	Manufacturer	Catalog number
P53	Oncogene	#OP03
P21	SantaCruz	#sc-6546
RB	BD Bioscience	#554136
GAPDH	Abcam	#8245
TUBULIN	SantaCruz	#sc-5286
VINCULIN	SantaCruz	#sc-5573
Swine anti-Rabbit HR	Dako	#P0217
Rabbit anti-Rat HRP	Dako	#P0450
Rabbit anti-Mouse HRP	Dako	#P0161

<u>ChIP-seq</u>

Name	Manufacturer	Catalog number
P53 antibody	Novocastra	#CM5P

Table S2: Reagents used in this study

Name	Manufacturer	Catalog number
ECL substrate	ThermoFisher	#32106
SuperScriptTM III Reverse Transcriptase	ThermoFisher	#18080093
Random primers (p(dN)6	Roche	#11034731001
iQ™ SYBR® Green Supermix	Bio-Rad	#1725006
Ribo-Zero Gold Kit	Epicentre Madison	#MRZG126
KAPA Stranded RNA-Seq Library Preparation Kit	KAPA	#8400
KAPA Hyper Prep Kit	KAPA	#KK8504
MinElute PCR Purification Kit	QIAGEN	#28006
RNeasy Mini Kit	QIAGEN	#74106
Lipofectamine-3000	ThermoFisher	#L3000015

Supplemental References

Dobin, A., Davis, C. A., Schlesinger, F., Drenkow, J., Zaleski, C., Jha, S., … Gingeras, T. R. (2012). STAR: ultrafast universal RNA-seq aligner. *Bioinformatics*, *29*(1), 15–21. https://doi.org/10.1093/bioinformatics/bts635

Li, H., & Durbin, R. (2009). Fast and accurate short read alignment with Burrows–Wheeler transform. *Bioinformatics*, *25*(14), 1754–1760. https://doi.org/10.1093/bioinformatics/btp324

Love, M. I., Huber, W., & Anders, S. (2014). Moderated estimation of fold change and dispersion for RNA-seq data with DESeq2. *Genome Biology*, *15*(12), 550. https://doi.org/10.1186/s13059-014-0550-8

Ramírez, F., Ryan, D. P., Grüning, B., Bhardwaj, V., Kilpert, F., Richter, A. S., … Manke, T. (2016). deepTools2: a next generation web server for deep-sequencing data analysis. *Nucleic Acids Research*, *44*(W1), W160–W165. https://doi.org/10.1093/nar/gkw257

Trapnell, C., Williams, B. A., Pertea, G., Mortazavi, A., Kwan, G., van Baren, M. J., … Pachter, L. (2010). Transcript assembly and quantification by RNA-Seq reveals unannotated transcripts and isoform switching during cell differentiation. *Nature Biotechnology*, *28*, 511. Retrieved from https://doi.org/10.1038/nbt.1621

Zhang, Y., Liu, T., Meyer, C. A., Eeckhoute, J., Johnson, D. S., Bernstein, B. E., … Liu, X. S. (2008). Model-based Analysis of ChIP-Seq (MACS). *Genome Biology*, *9*(9), R137. https://doi.org/10.1186/gb-2008-9-9-r137
